# Fluorocarbon Thin Films Fabricated using Carbon Nanotube/Polytetrafluoroethylene Composite Polymer Targets via Mid-Frequency Sputtering

**DOI:** 10.1038/s41598-017-01472-2

**Published:** 2017-05-03

**Authors:** Sung Hyun Kim, Cheol Hwan Kim, Woo Jin Choi, Tae Gon Lee, Seong Keun Cho, Yong Suk Yang, Jae Heung Lee, Sang-Jin Lee

**Affiliations:** 1 Chemical Materials Solutions Center, Korea Research Institute of Chemical Technology, Daejeon, 34114 Korea; 2 Center for Chemical Analysis, Korea Research Institute of Chemical Technology, Daejeon, 34114 Korea; 3 Department of Nano Fusion Technology, Pusan National University, Busan, 46241 Korea

## Abstract

Carbon nanotube/polytetrafluoroethylene composite polymer targets are proposed for use in the fabrication of fluorocarbon thin films using the mid-frequency sputtering process. Fluorocarbon thin films deposited using carbon nanotube/polytetrafluoroethylene composite targets exhibit an amorphous phase with a smooth surface and show a high water contact angle, optical transmittance, and surface hardness. X-ray photoelectron spectroscopy and Fourier transform infrared spectroscopy studies reveal that as the carbon nanotube concentration increased in the composite target, a carbon cross-linked structure was formed, which enhanced the film hardness and the modulus of the fluorocarbon thin film. Large-area fluorocarbon thin films with a substrate width of 700 mm were successfully fabricated by a pilot-scale roll-to-roll sputtering system using a carbon nanotube/polytetrafluoroethylene composite target.

## Introduction

Sputtered plasma polymer thin films using polymer targets have been widely studied and developed since the first report in the 1960s^1–12^. Among the various polymer targets, a polytetrafluoroethylene (PTFE) polymer target has been mostly used for depositing an organic thin film via a sputtering process^1–6, 12–58^. Fluorocarbon thin films deposited via radio-frequency (RF) sputtering using PTFE targets have many advantageous surface properties, such as hydrophobicity and super-hydrophobicity^30–36^, icephobicity^32^, oleophobicity^36^, high optical transmittance^37, 38^, dielectric^39^ and mechanical properties^40–47^, as well as antimicrobial^48^ characteristics. Thus, these thin films have recently garnered a substantial amount of attention in practical applications for flat panel displays, automobiles, fabrics^49^, membranes^50^, and high-frequency applications^51^.

Sputtered fluorocarbon thin films have been extensively studied by many groups. The Biederman group greatly contributed to developing RF-sputtered plasma polymer thin films^2–9, 12, 16, 18, 25, 30, 49, 53^. They reported notable results on the sputtering of various types of polymer targets under various gas conditions to form super-hydrophobic, nanocomposite thin films. The Faupel group focused on nanocomposite thin films formed by the methods of metal-polymer co-sputtering and multilayer processes^40, 45, 47, 51, 52^. The Iwamori group reported many research outcomes focusing on the optical and mechanical properties of RF-sputtered fluorocarbon thin films^22, 24, 33, 34, 38, 41, 42^.

RF sputtering is widely used when thin films are deposited using insulating materials^59^, but this method has a high cost and a low productivity and presents difficulties when applied to a large-area substrate. Recently, a mid-frequency (MF) sputtering method has been adopted to deposit insulating thin films using reactive sputtering with conductive targets instead of using RF sputtering^60–63^. The MF sputtering system typically using 20 to 80 kHz frequency generator that reduce signal reflection without additional matching box and improves the sputtering efficiency in a reactive sputtering process. With these advantages, many roll-to-roll sputtering systems have been adopted to produce flexible thin film devices using the MF sputtering process. However, most of the polymer targets are non-conductive materials that are difficult to apply to MF sputtering, for which most of the reported experiments have been performed using the RF sputtering method to deposit plasma polymer thin films.

In this study, we fabricated composite PTFE targets containing carbon nanotube (CNT) to impart an electrical conductivity to polymer targets and deposited fluorocarbon thin films by using MF sputtering with the composite targets. The influence of CNT in the composite targets on the properties of fluorocarbon thin films was investigated by determining the structural, surface, and optical properties of the films. In addition, we could fabricate a large-area fluorocarbon thin film with the CNT/PTFE composite target on polyethylene terephthalate (PET) substrates using a roll-to-roll sputtering system with a 700-mm substrate width.

## Results

**Figure 1:**
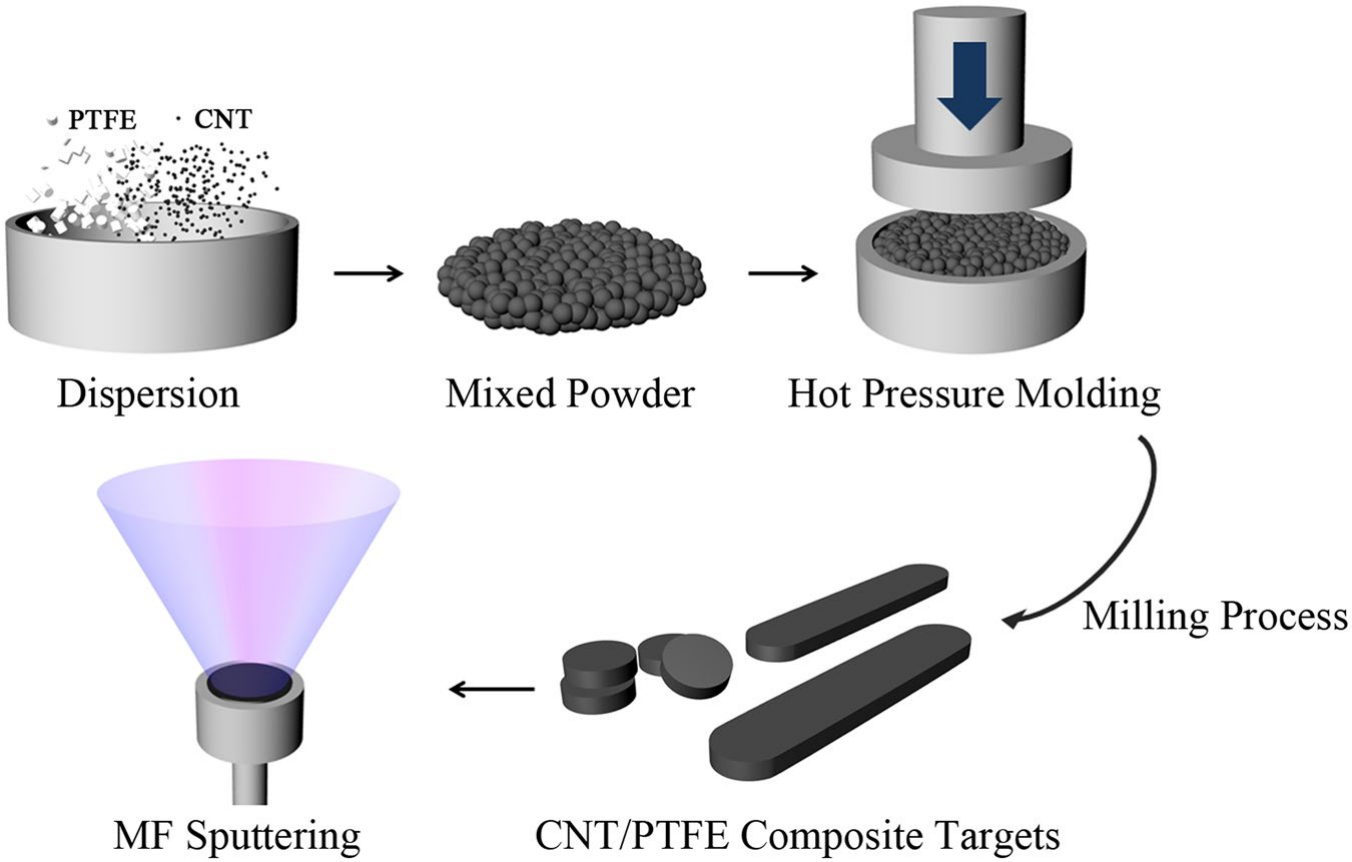
Schematic procedure for the fabrication of CNT/PTFE composite targets.

To impart electrical conductivity to the polymer target for MF sputtering, we mixed CNT powder with PTFE powder using CNT concentrations of 1, 3, 5, 10, and 15 wt%. Then, the conductive CNT/PTFE targets were shaped into 4-in disks. Figure 1 shows the schematic procedure of the CNT/PTFE composite target fabrication. All of the targets have a sheet resistance below 100 Ω/▫ to easily generate plasma with an MF power source. The sheet resistance of the target drastically decreased with increases in the CNT concentration up to 5 wt%. The decreasing rate of the sheet resistance was reduced over 5 wt%, and then, it finally reached the lowest value of 0.26 Ω/▫ at 15 wt% (Supplementary Figure S1, Supplementary Information). This low resistance of the polymer composite target allows the application of MF sputtering to fabricate the fluorocarbon thin films.

Fluorocarbon thin films of approximately 100-nm thickness using CNT/PTFE composite targets were fabricated using a test sputter system. The applied MF power was 100 W for the CNT concentrations of 1, 3, and 5 wt%, and 200 W of power was applied for the CNT 10 and 15 wt% targets. Supplementary Figure S2 shows a schematic of the test sputter system for depositing the fluorocarbon thin film using CNT/PTFE composite targets by MF sputtering.

**Figure 2:**
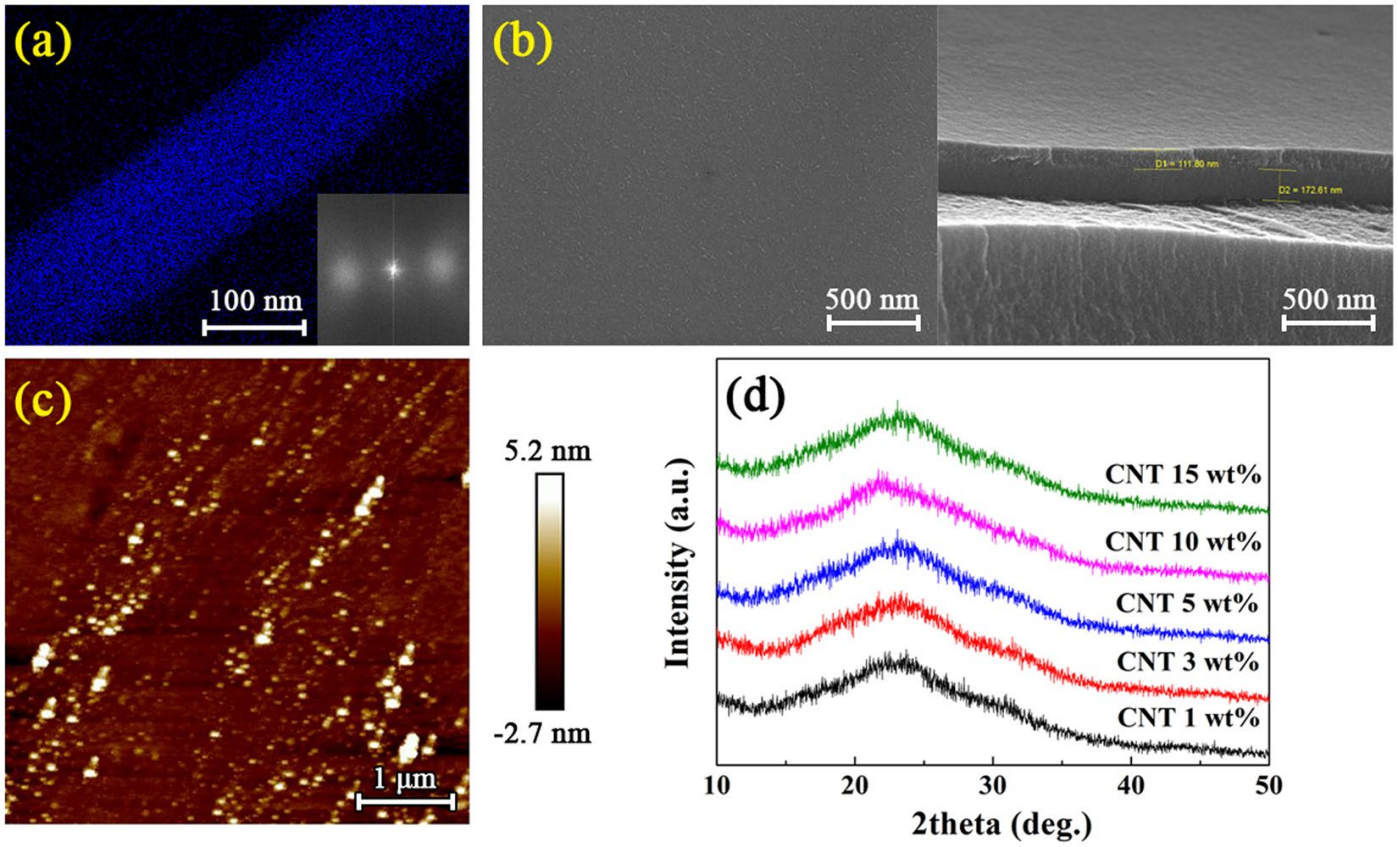
(**a**) TEM, (**b**) SEM, and (**c**) AFM images of the fluorocarbon thin films deposited using a 5 wt% CNT composite target, and (**d**) XRD patterns of the fluorocarbon films for various CNT/PTFE composite targets. The inset of (**a**) is the Laue diffraction image.

The cross-sectional transmission electron microscopy (TEM) image of the fluorine mapping of the 100-nm-thick fluorocarbon thin film deposited using the CNT 5 wt% target is shown in Fig. 2(a). From the TEM image, we confirm that the fluorocarbon thin film was successfully deposited onto the PET substrate using the CNT/PTFE composite target. The inset of Fig. 2(a) shows a Laue diffraction image of the fluorocarbon thin film. The sputtered fluorocarbon thin film obtained via a plasma polymer process has a typical amorphous structure. Thus, the fluorocarbon thin film shows high transparency and good flexibility. Figure 2(b) shows the cross-sectional scanning electron microscopy (SEM) image of the fluorocarbon thin film that has a uniform thickness and a smooth surface. This finding is consistent with the fluorocarbon thin film fabricated by sputtering PTFE, which appeared to be homogeneous, amorphous, and pinhole-free, as reported earlier^12, 14, 27^.

Atomic force microscopy (AFM) measurements were performed to further investigate the morphological properties of the fluorocarbon thin films from various CNT/PTFE composite targets. The results also indicate that the uniform surfaces and the surface roughness values (Ra) of the fluorocarbon thin films with CNT concentrations of 1, 3, 5, 10, and 15 wt% are 0.50, 2.04, 0.51, 0.51, and 2.64 nm, respectively, which are slightly lower values than the value of 3.35 nm resulting from the pristine PTFE target using RF sputtering. Many researchers have reported that sputtered PTFE on a Si substrate has an Ra in the range of 0.47~3.0 nm, depending on the process conditions^20, 22, 23, 26, 28^. Thus, our results are consistent with those previously reported. Figure 2(c) shows the AFM images of the fluorocarbon thin films fabricated using the CNT 5 wt% target.

To investigate in detail the structural properties of the fluorocarbon thin films fabricated using the CNT/PTFE composite targets, X-ray diffraction analysis was conducted. Figure 2(d) shows the amorphous patterns of the fluorocarbon thin films grown by MF sputtering using the CNT/PTFE composite targets, and these patterns are consistent with the Laue diffraction pattern in the inset of Fig. 2(a). These results indicate that no crystalline structures are present, considering that the X-ray peak position of the (100) for polycrystalline PTFE is located at approximately 18°; however, only amorphous phases are involved, as can be seen from the broad peaks at approximately 23° ^37, 64^. Biederman *et al*. proposed a molecular structural model of the fluorocarbon thin film prepared by RF sputtering, which suggests that the fluorocarbon thin film contains cross-linking structures that would enhance the formation of an amorphous structure^6, 38^.

**Figure 3:**
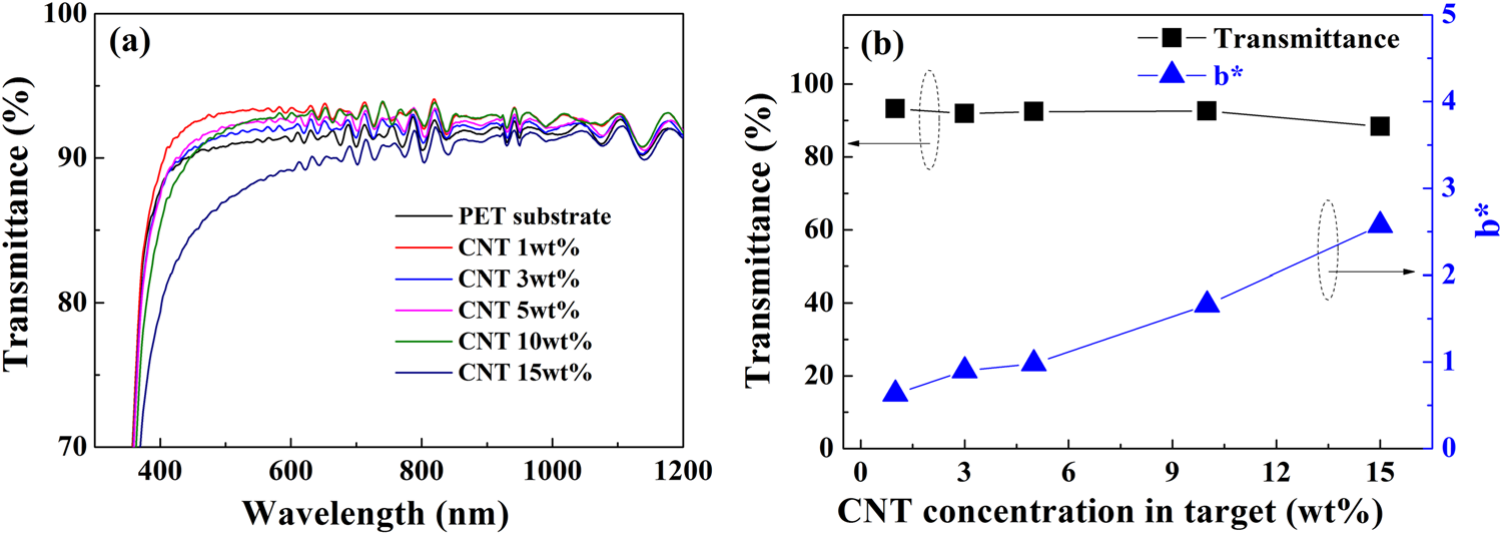
(**a**) The transmittance spectra in the wavelength range from 300 to 2400 nm, and (**b**) the optical transmittance at 550 nm and the yellow index of the fluorocarbon films deposited using CNT/PTFE composite targets.

In general, a sputtered fluorocarbon thin film shows high optical transparency because it has an amorphous structure^27^ and a low optical constant (n ≈ 1.38). Therefore, the transmittance of a fluorocarbon thin film deposited onto a PET film substrate could be higher than that of the PET substrate^37^. Figure 3(a) shows the optical transmittances of the fluorocarbon thin films on a PET substrate in the wavelength range between 300 and 2400 nm as a function of CNT concentration in the CNT/PTFE composite targets. The transmittance of the fluorocarbon coated on the PET substrate showed a higher value than those without a coating ranging from 300 to 1000 nm for CNT concentrations of 1~5 wt% in contrast to a low transmittance in the UV region as reported previously^37^. This result indicates that the transmittance can be enhanced due to the fluorocarbon thin film, which has a relatively low optical constant. However, the optical transmittance in the visible range decreases with increasing CNT concentration over 10 wt%. It is considered that the optical constants change with increasing carbon concentrations. Figure 3(b) shows the variation of the optical transmittances at a wavelength of 550 nm and the yellow indices (b*) calculated by ASTM E313 from spectrophotometric data of the fluorocarbon thin films on a PET substrate as a function of the CNT concentration in the CNT/PTFE composite targets. b* is an index of the color characteristics of the film and indicates the degree of yellowish^65^. The maximum optical transmittance of the fluorocarbon thin films with a CNT concentration of 1 wt% is 93.27% at a 550 nm wavelength, and the minimum optical transmittance with a CNT concentration of 15 wt% is 88.36%. The yellow index also increases with increasing CNT concentration in the composite target. The yellow color is common to all polymers prepared in a glow discharge because of the appreciable molecular damage inflicted on the cross-linked polymer^1, 12^. These results indicate that a highly transparent and low yellow index fluorocarbon thin film can be fabricated by MF sputtering using CNT/PTFE composite targets with a low CNT concentration.

**Figure 4:**
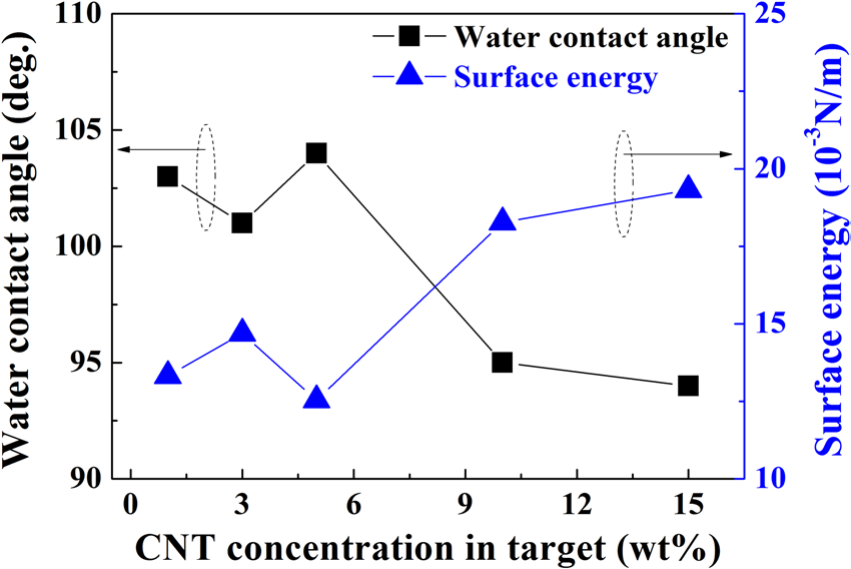
Water contact angles and calculated surface energies by Girifalco-Good-Fowkes-Young method of the fluorocarbon thin films as a function of the CNT concentration of the target.

Figure 4 shows the water contact angle and the surface energy of the fluorocarbon thin films as a function of the CNT concentration of the target. We observe that the water contact angle has a relatively constant value of 101~104° for a CNT concentration of 5 wt%, but it slightly decreases with CNT concentrations over 10 wt% because of the increasing carbon ratio in the fluorocarbon thin film. We calculated the surface energy of the fluorocarbon thin films deposited using the CNT/PTFE composite targets via the Girifalco-Good-Fowkes-Young (GGFY) method (Supplementary Table S1, Supplementary Information)^66^. As shown in Fig. 4, the surface energies of the fluorocarbon thin films are 13.32, 14.69, 12.54, 18.27, and 19.32 × 10^−3^ N/m, and the water contact angles are 103, 101, 104, 95, and 94° with increasing CNT concentrations in the CNT/PTFE targets.

**Figure 5:**
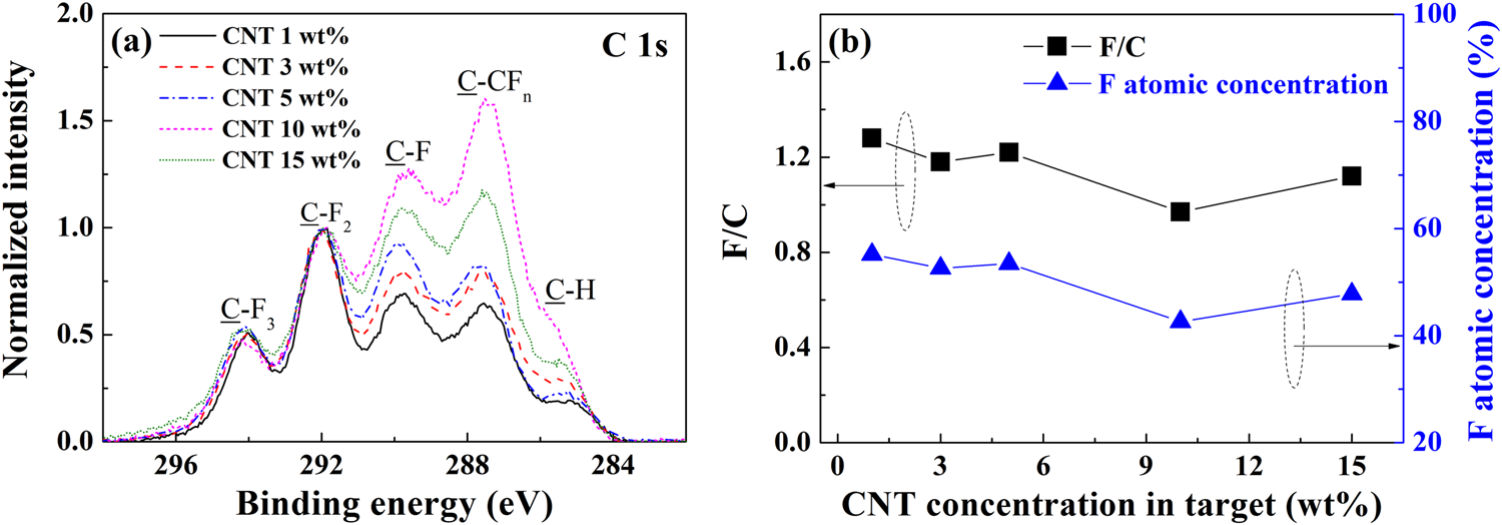
Normalized XPS spectra of (**a**) C-1s and (**b**) the calculated F/C ratio for the fluorocarbon thin films deposited using CNT/PTFE composite targets.

To obtain more information about the influence of the CNT concentration on the composite target during deposition, we investigated the molecular structures of the fluorocarbon thin films using X-ray photoelectron spectroscopy (XPS). Figure 5 shows the XPS C-1s core level spectra and the F/C ratios of the fluorocarbon thin films deposited using the CNT/PTFE composite targets. In the sputtering process, CNT is not included in itself in the plasma polymer fluorocarbon thin film, but is decomposed by carbon and recombined with fluorine to exist as a fluorocarbon. The C-1s spectra show broad peaks with 5 prominent Gaussian deconvolution peaks that have the following customary assignments based on the XPS study for fluorocarbon thin films: 294.0 eV (CF_3_), 292.0 eV (CF_2_), 289.8 eV (CF), and 287.5 eV (C-CF_n_). The peak at 285.2 eV represents hydrocarbon (-CH_2_-) surface contamination^16, 18, 29, 67–70^. For a comparison of the spectral features, the peaks are normalized to the peak corresponding to 292.0 eV (CF_2_). Figure 5(a) indicates that the intensities of the C-F and C-CF peaks increase as the CNT concentration increases from 1 to 15 wt%, while the fluorine contents decrease. These results indicate that a highly cross-linked structure is formed as the carbon contents are increased^38, 42, 50^. F-1s peaks appear near 689.2 eV, and the intensities of the F-1s peaks of all fluorocarbon thin films have similar values (Supplementary Figure S3, Supplementary Information).

The F/C ratios of the thin films were calculated from the relative intensities of the deconvolution peaks in the C-1s spectra (Supplementary Table S2, Supplementary Information). Many researchers have reported F/C ratios ranging from 0.78~1.60 ^18, 29, 31, 38, 42, 47^. Among these researchers, Golub *et al*. determined a highly reliable F/C ratio of 1.49 for sputtered fluorocarbon thin films from 14 repeated measurements^18^. Figure 5(b) shows that the F/C ratios of the fluorocarbon thin films from CNT concentrations of 1 to 15 wt% have a decreasing trend as the CNT concentration increases from 1.28 to 1.12. We found that the decrease in the F/C ratio for the thin films fabricated with a higher CNT concentration target is caused by a decrease of the carbon-fluorine coordination in the fluorocarbon thin film. Correspondingly, there is a decrease in the surface energy and in the optical transmittance when a high CNT concentration target is used.

**Figure 6:**
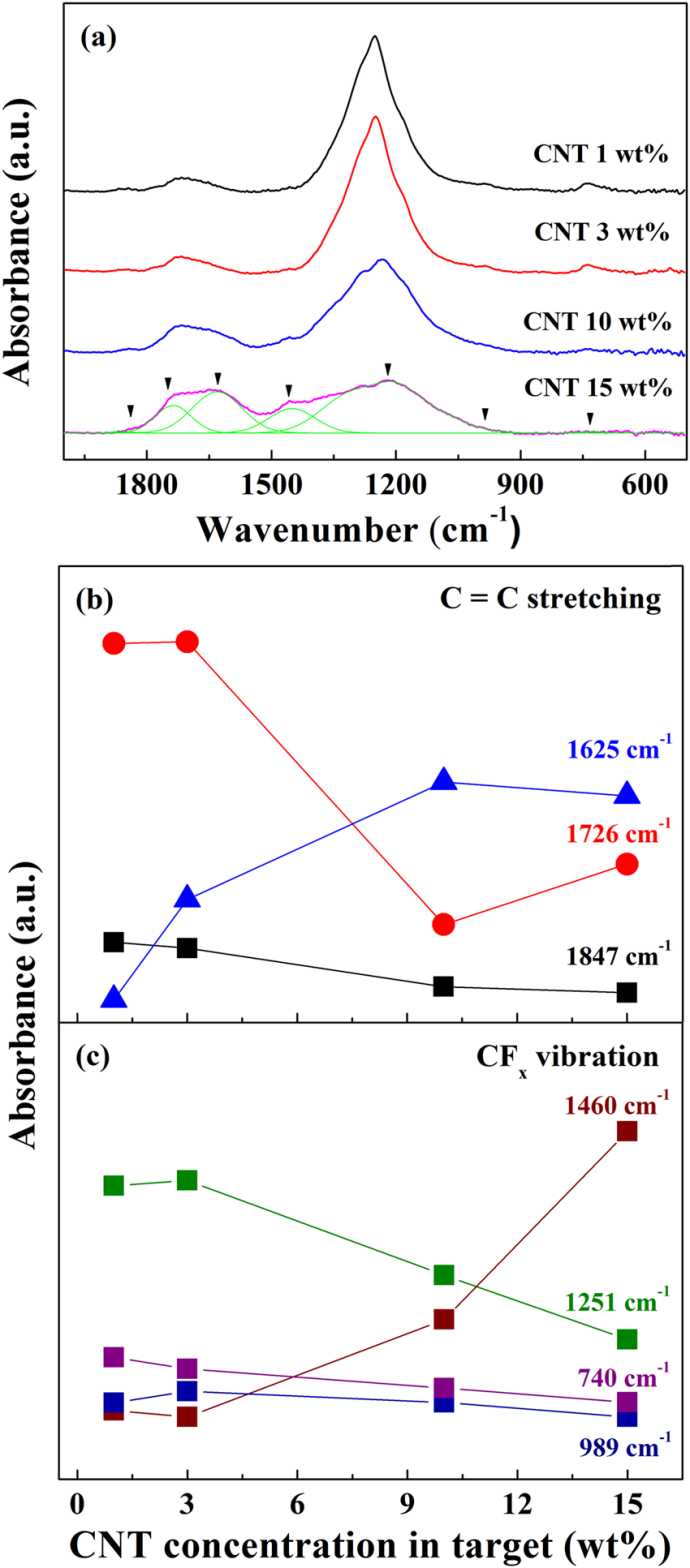
(**a**) FT-IR reflectance absorption spectra of the fluorocarbon thin films. Intensity changes in the reflectance absorption of (**b**) C=C stretching and (**c**) CF_x_ vibration modes. To compare easily, 1251 cm^−1^ (green) was 5 times scaled-down.

As observed in the change of binding energies from XPS measurements, we also observed changes in the phonon modes in the FT-IR spectra. Figure 6(a) shows the normalized reflectance absorption spectra of the fluorocarbon thin film with a CNT concentration between the wavenumbers of 500 and 2000 cm^−1^. The C=C stretching and CF_x_ vibrations overlap in this region, and Gaussian curves are used to separate individual peaks in the regions of 1400~2000 cm^−1^ (C=C stretching) and 500~1800 cm^−1^ (CF_x_ vibrations). Each peak is assigned as follows: F_2_C=CF stretching at 1847 cm^−1^, F_2_C=C stretching at 1726 cm^−1^, HFC=C stretching at 1625 cm^−1^, CF_2_ asymmetric stretching at 1460 cm^−1^, CF_2_ symmetric stretching at 1251 cm^−1^, and amorphous PTFE or CF_3_ vibrations at 989 cm^−1^ and 740 cm^−1^ ^21, 33, 71^. The C=C stretching peak shows a red shift when there is less fluorine^71^. During the sputtering process, some fractions of fluorine are shared and move from PTFE to the CNTs; therefore, the peak intensity at 1625 cm^−1^ increases, and those at 1847 and 1726 cm^−1^ decrease when the CNT ratio increases (Fig. 6(b)). The same absorption results are observed in the CF_x_ vibration region. Figure 6(c) shows that the peak intensities at 989 and 740 cm^−1^ decrease, but the intensity at 1460 cm^−1^ increases as the CNT concentration increases. However, the peak intensity at 1251 cm^−1^, which we assign as CF_2_ symmetric stretching, decreases. According to V. Stelmashuk *et al*., the C-C and CF_x_ stretching vibrations, such as those of CF_3_–, –CF_2_–, CF_3_CF_2_–, CF_2_=CF– and CF_2_=C<, overlap in this region^22^. The extinction coefficient of each vibrational mode is unknown. The total peak area of the C=C stretching and CF_x_ vibration regions decreases as the CNT concentration increases. Therefore, we assume that the less fluorinated carbons have a lower extinction coefficient than the highly fluorinated carbons, and as a result, the peak intensity at 1251 cm^−1^ decreases.

**Figure 7:**
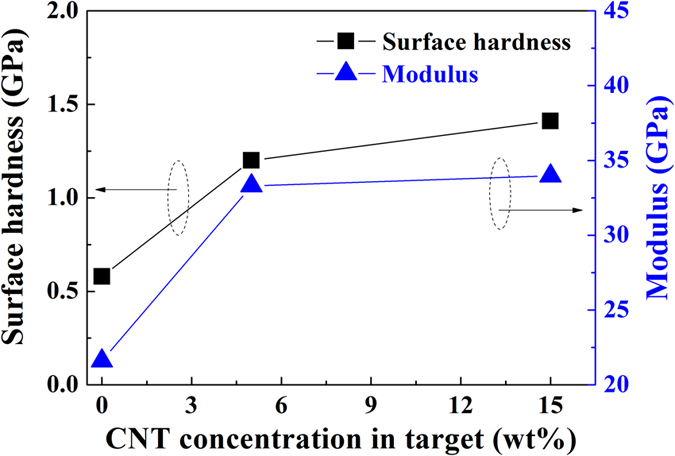
Surface hardness and modulus of the fluorocarbon thin film as a function of the CNT concentration in the composite target.

The XPS and FT-IR results indicate that a highly cross-linked carbon structure is formed as the CNT concentration increases in the CNT/PTFE composite target, and this structure decreases the optical transmittance and surface energy. However, the cross-linked carbon structure enhances the hardness and modulus of the fluorocarbon thin film. Figure 7 shows the surface hardness and modulus data of the fluorocarbon thin film deposited using CNT/PTFE composite targets. For comparison, the RF sputtering of pristine PTFE (with a CNT concentration of 0 wt%) was conducted. The surface hardness of the fluorocarbon thin film increases by as much as double from 0.58 GPa (CNT 0 wt%) to 1.20 GPa (CNT 5 wt%) when CNT are incorporated into the PTFE composite target, and the modulus increases from 21.6 GPa (CNT 0 wt%) to 33.3 GPa (CNT 5 wt%). Moreover, the surface hardness and modulus further increase with the CNT concentration in the CNT/PTFE composite targets. From these results, we confirm that a high hardness fluorocarbon thin film with high hydrophobicity and transparency can be fabricated via MF sputtering of the CNT/PTFE composite target.

**Figure 8:**
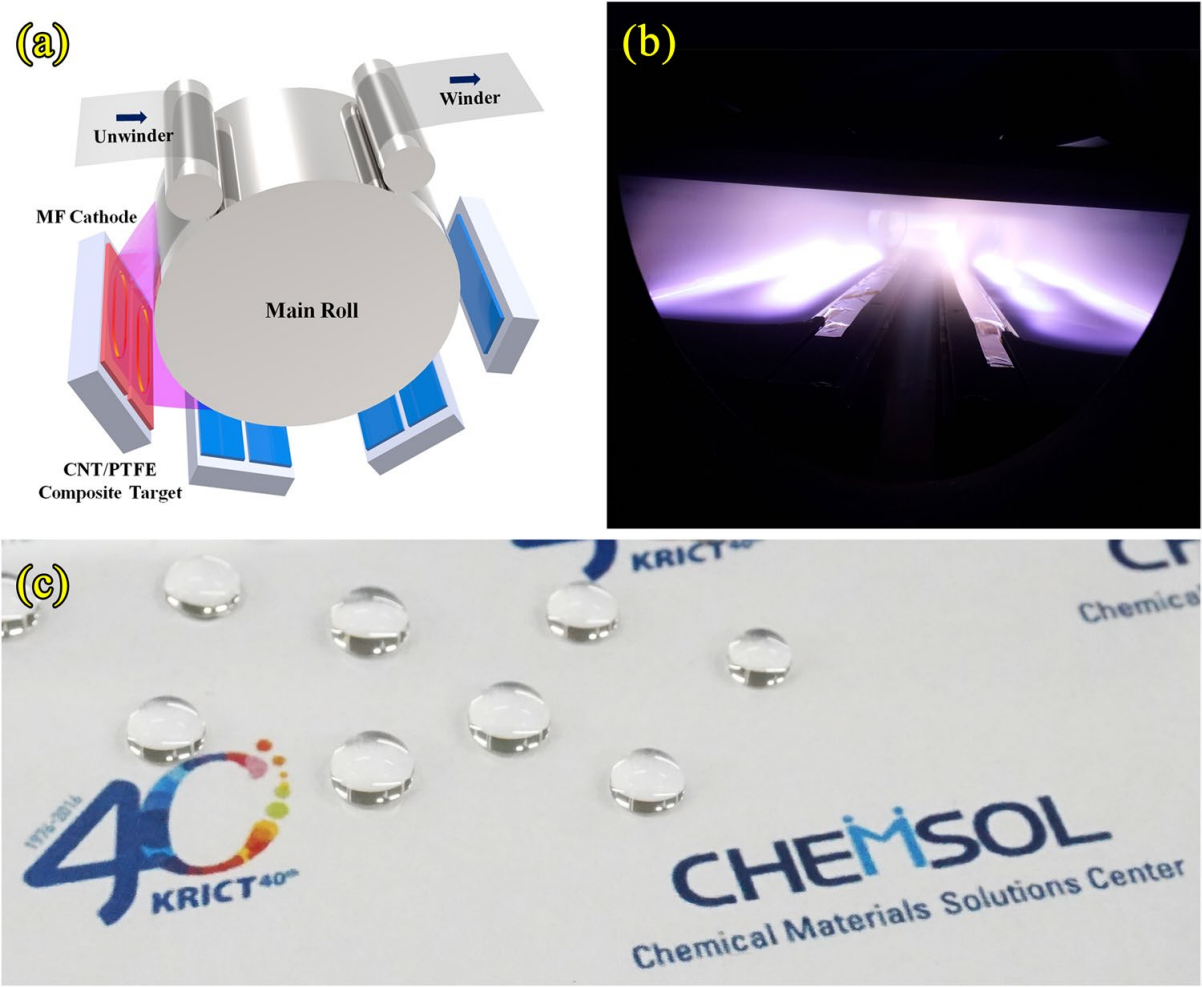
Images of (**a**) an enlarged schematic of the sputter chamber, (**b**) the sputtering plasma, and (**c**) the hydrophobic and transparent fluorocarbon thin film. CHEMSOL logo in the Fig. 8(c) was used under permission of the Chemical Materials Solutions Center.

Large-area fluorocarbon thin films with a thickness of 30 nm were deposited on roll-type 100-μm-thick hard-coated PET substrates (SH60, SKC) using a pilot scale roll-to-roll sputtering system with a 700-mm film width. Figure 8(a) shows an enlarged image of the sputter chamber. We fabricated a rectangular-shaped CNT/PTFE composite target (CNT 5 wt%:PTFE 95 wt%) with dimensions of 960 × 150 × 6 mm^3^ using a high-temperature compression molding method. The large-area fluorocarbon thin films formed using composite targets with 5 wt% CNT were deposited under an Ar atmosphere at room temperature. The applied MF power was 2.5 kW (1.74 W/cm^2^), and the Ar gas feeding rate was 400 sccm. The line speed of a roll-type PET substrate was 1 m/min in the sputtering process. As shown in Fig. 8(b), no arc, spark, or flicker distortion of the plasma or target occurred. We fabricated a large-area fluorocarbon thin film that was over 100-m long. The large area fluorocarbon thin film shows a high uniformity of thickness (under 5%), a high transmittance of 92%, and a hydrophobic surface that has a water contact angle of 110°. Figure 8(c) shows the hydrophobic and transparent properties of the large-area fluorocarbon thin film fabricated using the pilot scale roll-to-roll sputtering process. From these results, we confirm that large-area fluorocarbon thin films that have a high water repellency, optical transparency and mechanical hardness can be fabricated via roll-to-roll sputtering using MF power for mass production.

## Conclusion

In conclusion, CNT/PTFE composite targets with CNT concentrations of 1, 3, 5, 10, 15 wt% were fabricated for depositing a fluorocarbon thin film. CNT impart electrical conductivity to the polymer target, which allows the application of the MF sputtering process to fabricate plasma polymer thin films possessing various characteristics. TEM, SEM, AFM and XRD studies of the fluorocarbon thin films reveal an amorphous structure possessing a good uniformity of nanometer-level thickness and a smooth surface. Because of the amorphous structure and low optical constant, the fluorocarbon thin film deposited on a PET substrate shows a higher optical transmittance than that of the PET substrate. The water contact angle measurement indicates that the sputtered fluorocarbon thin film has a good hydrophobic property and a low surface energy. However, the optical and surface properties are reduced as the CNT concentration increases in the composite target. Furthermore, we observe from the XPS and FT-IR analysis that a carbon cross-linked structure is formed as the CNT concentration increases, resulting in a good mechanical hardness and modulus in the fluorocarbon thin film obtained from the CNT/PTFE composite targets.

Moreover, a large-area fluorocarbon thin film with a 700-mm substrate width was fabricated using a pilot-scale roll-to-roll sputtering system. The film shows excellent hydrophobicity and transparency with a high hardness and good thickness uniformity. These results demonstrate that a large-area fluorocarbon thin film with various functions, such as hydrophobicity, transparency, and high hardness, can be fabricated using a MF sputtering system for various applications, such as flexible displays, automobiles, fabrics and electronic devices.

## Methods

### Preparation of CNT/PTFE Composite Target

Multiwall CNT powder (HANOS CM-280, Hanwha Chemical) and PTFE powder (A7-J, Dupont Mitsui) were pre-mixed with CNT:PTFE weight ratios of 1:99, 3:97, 5:95, 10:90, and 15:85. The CNT/PTFE composite polymer targets were fabricated using a high-temperature compression molding method. Disk-shaped CNT/PTFE targets (CNT 1, 3, 5, 10, and 15 wt%) with a 4-in diameter and 6-mm height were then prepared using the milling process.

### Fluorocarbon thin film fabrication

Test samples for characterizing electrical, optical, mechanical and morphological properties were fabricated using a test sputter system. We used the CNT/PTFE 4-in diameter composite targets and the hard-coated PET (Kimoto) film substrate with a size of 10 × 10 cm^2^ and a thickness of 188 μm, as well as a glass substrate (Marienfeld-Superior) with a size of 2.6 × 7.8 cm^2^. The sputtering chamber was evacuated using a cryopump backed with a mechanical pump. The base pressure was approximately 5 × 10^−5^ Pa. Pure argon (Ar) was used as the sputtering gas, and the flow rate was controlled by a mass flow controller.

The MF power was applied at a fixed value of 100 W (1.24 W/cm^2^) for CNT concentrations of 1~5 wt% and 200 W (2.48 W/cm^2^) for CNT concentrations of 10~15 wt% because the sputtering yield was very low at 100 W for composite targets with a high CNT concentration.

### Large-area sample fabrication using roll-to-roll sputtering

A large-area fluorocarbon thin film was deposited on the 188-μm-thick, 700-mm-wide PET film (SH-40, SKC) substrate using a pilot-scale roll-to-roll sputtering system. This system consists of three modules: the unwinder, winder, and main sputtering modules. The main sputtering module contains four separate compartments with three MF dual cathodes and one DC cathode for depositing multilayer films. In this experiment, we used 1 MF cathode for the deposition of the fluorocarbon thin film with a CNT/PTFE composite target.

Before the deposition was performed, the pretreatment of the substrate film was carried out using a heater and the Ar/O_2_ ion plasma to remove surface contamination and to improve the adhesion between the fluorocarbon thin film and the PET substrate in the unwinder chamber. A film pretreatment was performed by heating the film to 300 °C (the film surface temperature is approximately 65 °C under the condition of a line drive speed of 1 m/min) and exposing it to an Ar/O_2_ ion plasma at 400 W while passing the film through the unwinder chamber. Subsequently, a large-area fluorocarbon thin film was continuously deposited onto the roll-type PET substrate via MF sputtering with a CNT 5 wt%/PTFE 95 wt% target.

### Characterizations of Sputtered Fluorocarbon Thin Films

The sheet resistance measurements of the CNT/PTFE composite targets were performed at room temperature using a standard four-point probe technique (MCP-T610, Mitsubishi Chemical Analytech). The structural and morphological properties of the fluorocarbon thin films were investigated using TEM (TECNAI G2 T-20S, FEI Co.), field-emission scanning electron microscopy (FE-SEM, MIRA3 LMU FEG, Tescan), AFM (Nanoscope V, Bruker), and XRD (SmartLab, Rigaku). The optical transmittances were measured in the wavelength range of 300~2400 nm using an optical spectrometer (U-4100, Hitachi). The surface energy of the fluorocarbon thin film was calculated by measuring the water contact angle with a contact angle analyzer (PHOENIX 300 touch, Surface Electro Optics). The amount of water droplets was 2 μL and the captured images were systematically analyzed in the contact angle measurement. The chemical structure was analyzed using Fourier transform infrared spectroscopy (FT-IR, VERTEX 80 v, Bruker) and X-ray photoelectron spectroscopy (XPS, AXIS NOVA, Kratos). The film surface hardness and modulus were measured using a nanoindentor (Nanoindentor XP, Agilent). 100 nm-thick fluorocarbon thin films were deposited on silicon wafers to measure the nanohardness and the changes of hardness by indentation depth with incremental force from 2 μN to 3 mN were measured (continuous multicycle indentation method). For comparison between samples, the hardness measurements at an indentation depth of 20 nm were analyzed.

## Electronic supplementary material

Supplementary Information

Supplementary Video
