# Supplementary material for: Fluorocarbon Thin Films Fabricated using Carbon Nanotube/Polytetrafluoroethylene Composite Polymer Targets via Mid-Frequency Sputtering
Source: Sci Rep. 2017 May 3;7:1451. doi: 10.1038/s41598-017-01472-2 (PMC5431166; doi:10.1038/s41598-017-01472-2)
Supplement: Supplementary file 1 — Supplementary Information [file 41598_2017_1472_MOESM1_ESM.pdf]

Supplementary Information

Fluorocarbon Thin Films Fabricated using Carbon Nanotube/Polytetrafluoroethylene Composite Polymer Targets via Mid-Frequency Sputtering

Sung Hyun Kim, Cheol Hwan Kim, Woo Jin Choi, Tae Gon Lee, Seong Keun Cho, Yong Suk Yang, Jae Heung Lee and Sang-Jin Lee**

Sung Hyun Kim, Cheol Hwan Kim, Woo Jin Choi, Seong Keun Cho, Jae Heung Lee, Sang-Jin Lee

Chemical Materials Solutions Center, Korea Research Institute of Chemical Technology, Daejeon 34114, Korea

Tae Gon Lee

Center for Chemical Analysis, Korea Research Institute of Chemical Technology, Daejeon 34114, Korea

Sung Hyun Kim, Yong Suk Yang,

Department of Nano Fusion Technology, Pusan National University, Busan 46241, Korea

* jahlee@krict.re.kr, leesj@krict.re.kr (co-corresponding)

Supplementary Figures

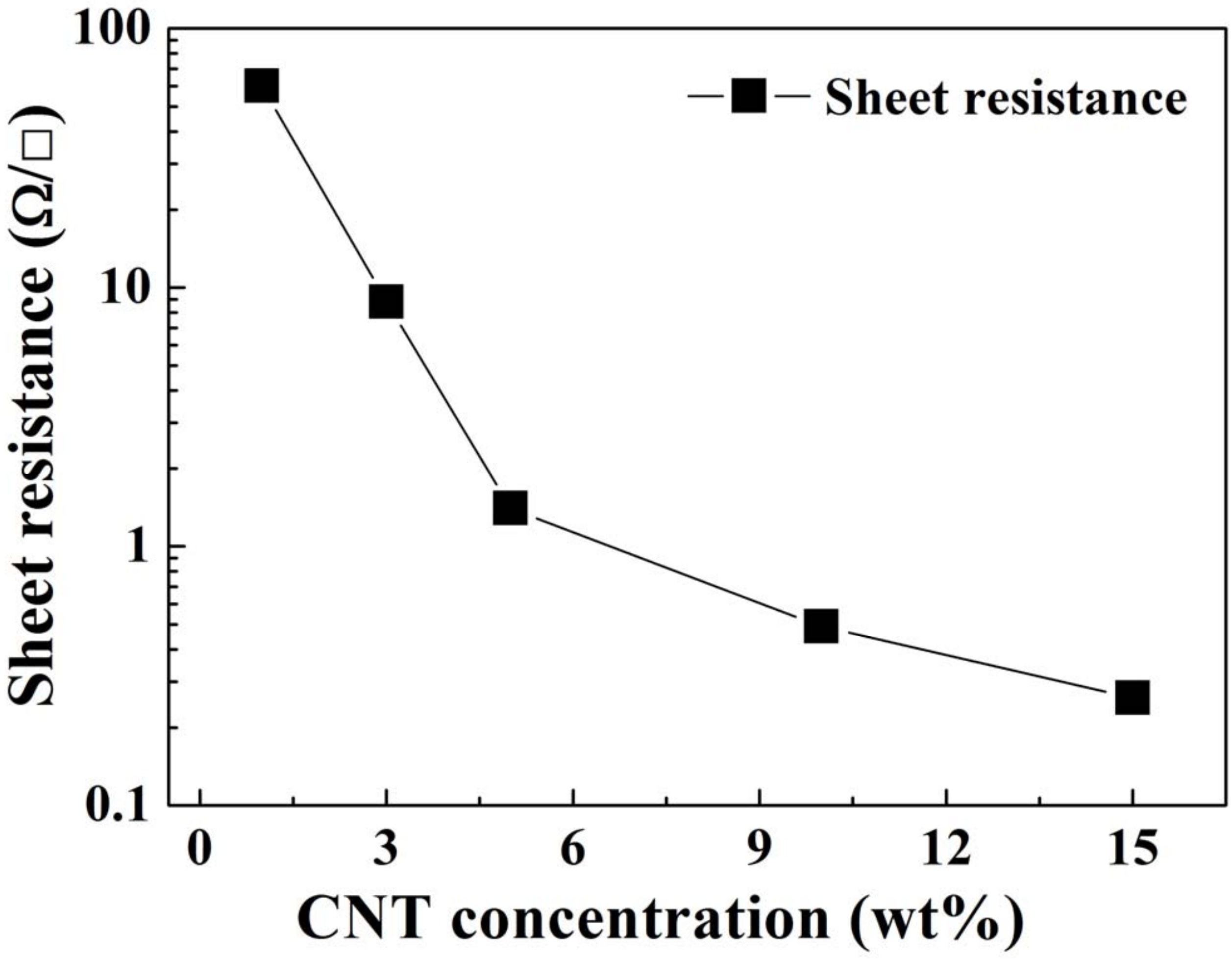

Supplementary Figure S1. Sheet resistances of CNT/PTFE composite targets as a function of CNT concentration.

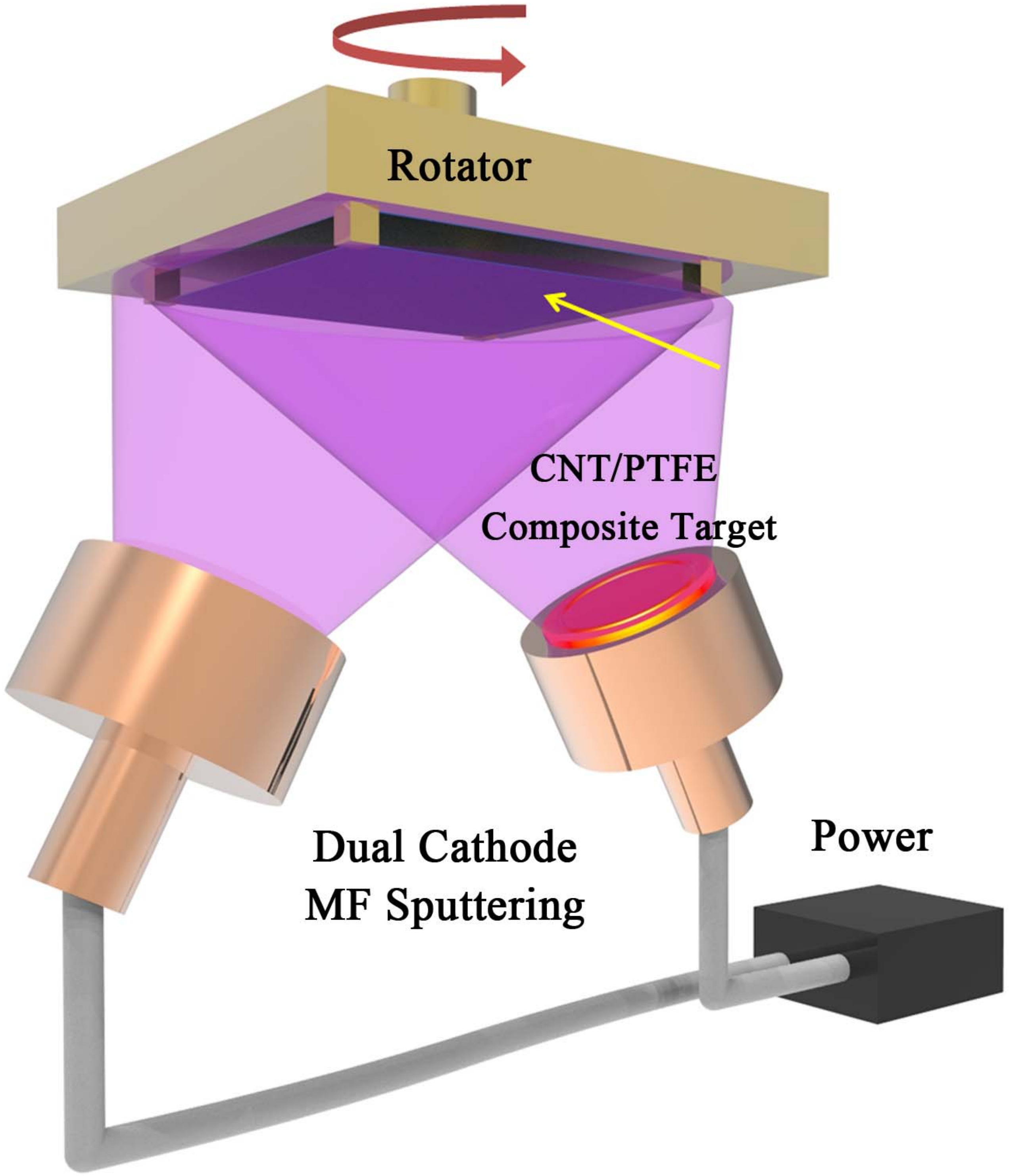

Supplementary Figure S2. Schematic of the test sputter system for depositing the fluorocarbon thin film using a CNT/PTFE composite target.

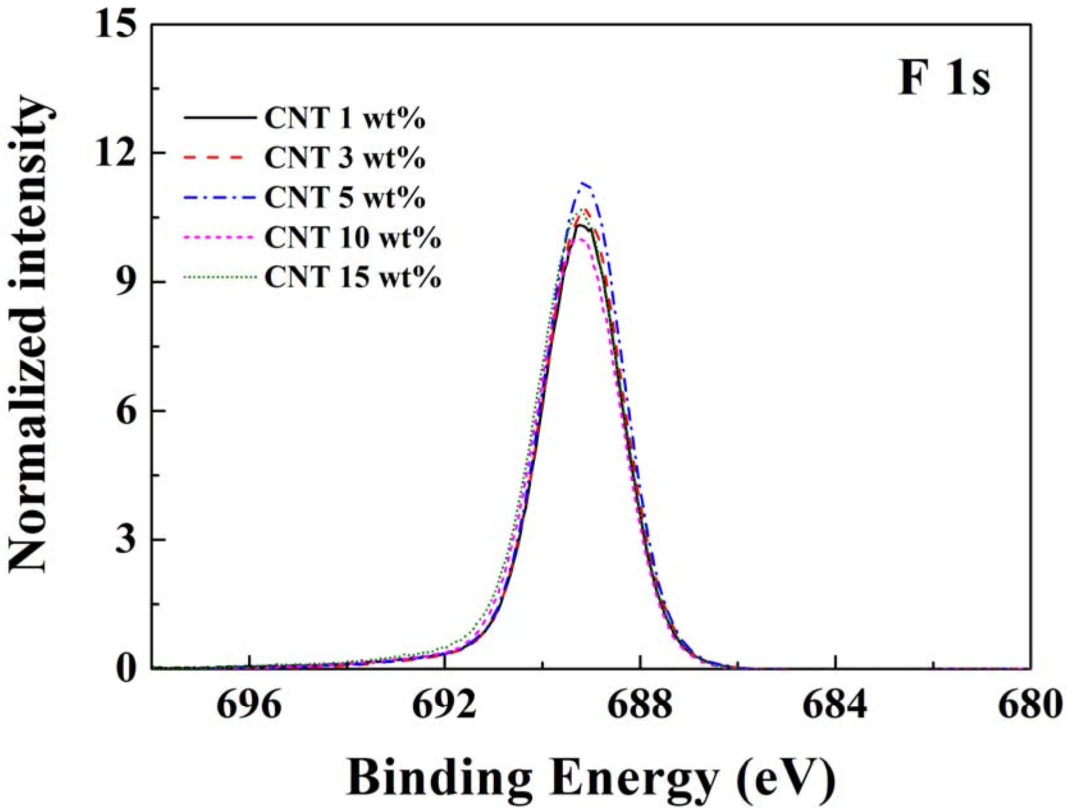

Supplementary Figure S3. Normalized XPS spectra of F-1s for the fluorocarbon thin films deposited using CNTs/PTFE composite targets.

Supplementary Tables

Supplementary Table S1. Surface energy and water contact angle values of fluorocarbon thin films

CNT contents (wt%)	Surface energy (10^{-3}N/m)	Contact angle (°)
1	13.320	103
3	14.687	101
5	12.538	104
10	18.271	95
15	19.316	94

Supplementary Table S2. Calculated F/C ratio and fluorine atomic concentration from XPS spectra

CNT wt%	F/C ratio	F atomic concentration (%)
**1**	1.28	55.17
**3**	1.18	52.60
**5**	1.22	53.46
**10**	0.97	42.61
**15**	1.12	47.78

Supplementary Video

Supplementary Video. Video shows hydrophobic and transparnet properties of large area fluorocarbon thin film on PET film fabricated by using the pilot scale roll-to-roll sputter.
